# Preparation and Characterization of Potato Starch Film with Various Size of Nano-SiO_2_

**DOI:** 10.3390/polym10101172

**Published:** 2018-10-22

**Authors:** Rongfei Zhang, Xiangyou Wang, Meng Cheng

**Affiliations:** Department of Food Science and Engineering, Shandong University of Technology, Zibo 255000, China; shenggongrongfei@163.com (R.Z.); chengmeng0110@163.com (M.C.)

**Keywords:** potato starch films, nano-SiO_2_, antibacterial property, size

## Abstract

The various sizes (15, 30, 80, and 100 nm) of nano-SiO_2_/potato starch films were synthesized and characterized. The gas permeability, antibacterial properties, and mechanical properties of the films were evaluated to their potential for application as food packaging materials. Results indicated that the 100 nm nano-SiO_2_ was well dispersed in the starch matrix, which induced an active group on the surface of 100 nm nano-SiO_2_ adequately combined with starch macromolecule. The water resistance and mechanical properties of the films were improved with the addition of nano-SiO_2_. Notably, resistance to ultraviolet and thermal aging was also enhanced. The nano-SiO_2_/potato starch films were more efficient against *Escherichia coli* (*E. coli*) than *Staphylococcus aureus* (*S. aureus*). Remarkable preservation properties of the films packaging the white mushrooms were obtained, with those of the 100 nm films considered superior. This study can significantly guide the rational choice of the nano-SiO_2_ size to meet the packaging requirements of various agricultural products.

## 1. Introduction

The use of starch films shows a rational use of natural resources and reduces environmental pollution. Starch films present many advantages, such as low cost, renewability, biodegradability, and ease of processing. The starch films are exploited as a promising kind of commercial preservation films for extending the shelf of food. Different kinds of starch have been widely used to prepare the films [1,2,3,4]. Therein, potato starch presents better film-forming properties than that of other cereal starch prepared films [5]. However, the use of potato starch with 80% large macromolecules of branched amylopectin presents several disadvantages in packaging-poor moisture sensitivity, poor mechanical properties, and poor solubility [6]. Therefore, potato starch films exhibit low food preservation efficiency.

To enhance the properties of potato starch films, the surface hydroxyl groups and small size of the nanoparticles were considered. The superiority of potato starch-based nanocomposite films is attributed to the uniform dispersion of nanoparticles in the potato starch and hydrogen bonding with starch molecules through surface hydroxyl groups [7]. These qualities allow the enhancement of permeability and mechanical properties. According to related studies, the size, shape, and hydrophily of modified nanoparticles could be adjusted to improve the properties of films [8]. Several nanoparticles, such as nano-ZnO, nano-TiO_2_, nano-SiO_2_, and nano-clay, have been evaluated as potential nanomaterial [6,9,10,11,12]. Among these nanomaterials, nano-SiO_2_ exhibits the greatest potential because of its superior properties, including an extremely porous structure and high surface activity. Moreover, nano-SiO_2_ is chemically inert and biocompatible, and prevents bacterial attack in vitro and in vivo [13]. Therefore, nano-SiO_2_ polymer films can maintain acceptable levels of sensorial and physiological qualities and a longer storage life for food. Song et al. (2016) [14] found that chitosan/nano-silica coating could reduce sugars and induce high antioxidant enzyme activities to improve chilling tolerance in loquat fruit. The chitosan/nano-silica coating could also extend the storage period of fresh longan fruits [15]. However, most studies on nano-SiO_2_ polymer films focused on the effect of nano-SiO_2_ dosage on preservation properties [16]. Meanwhile, the hydrophilic surface of nano-SiO_2_ in the films was modified. Despite such a modification, the original size of nano-SiO_2_ limited the modified efficiency of the films. Therefore, modification costs should be reduced, and the original size of nano-SiO_2_ should be controlled. Several studies found that nano-SiO_2_ dispersion facilitated synthesis in situ [17]. The effect of nano-SiO_2_ size on its dispersion in films has been rarely reported. In addition, nano-SiO_2_ exhibits a reflectivity of 70%–80% for ultraviolet light (wavelength number ≤ 490 nm), which is incorporated into the potato starch film to resist ultraviolet aging and thermal aging [16,18]. Meanwhile, nano-SiO_2_ could also induce cytotoxicity and protein alteration in cells [19]. The cytotoxicity of nano-SiO_2_ was influenced by its size. The films with nano-SiO_2_ of different sizes might exert certain effects on microorganisms. Studies are rarely reported on the antibacterial property of nano-SiO_2_ polymer films with nano-SiO_2_ of different sizes.

The particle size is a key factor for affecting a number of technical performances and practical applications of nano-SiO_2_. For the integration of nano-SiO_2_ into polymer films, the separation of film particles from one another, in addition to particle size, is important. The aggregates could reduce the properties of the films [20]. The particle size and uniformity of nano-SiO_2_ in the films significantly affect the performance of the films. The demand for monodisperse nano-SiO_2_ particles with a narrow particle size distribution is increasing. However, as the nano-SiO_2_ size decreases, the surface energy gradually increases. When the particle size is 25 nm or less, the absolute value of the surface potential of the particles and the stability of the nano-SiO_2_ colloidal system are reduced; in addition, electrostatic repulsion between the particles is reduced. Consequently, agglomeration occurs between nano-SiO_2_ particles to decrease its monodispersity. Studies on the effect of suitable particle size on the performance of nano-SiO_2_/potato starch films have thus far been rarely reported. Most studies mainly aim to enhance the properties of the film by modifying the nano-SiO_2_ particles [21] and adopting novel methods [22], which is neither cost-efficient nor ecologically friendly.

In the present study, nano-SiO_2_/potato starch films of different sizes (15, 30, 80, and 100 nm) were synthesized by solution casting and characterized by X-ray diffraction, Fourier transform infrared spectroscopy, and scanning electron microscopy. The effects of gas permeability, water resistance, tensile strength, thermal stability, antibacterial properties, and preservation properties of the films on white mushrooms (*Agaricus bisporus*) were evaluated for their potential application as food packaging materials. To obtain the best properties, the optimal size of the nano-SiO_2_ particle was determined. This study guides the selection of the appropriate size of nano-SiO_2_ used directly in films to reduce processing costs or to combine with a dispersive method in order to improve the properties of the films.

## 2. Materials and Methods

### 2.1. Materials

Nano-SiO_2_ (15, 30, 80, and 100 nm) was purchased from Beijing Obo Biotechnology Co., Ltd. (Beijing, China). Potato starch was obtained from Beijing Aoboxing Bio-tech Co., Ltd. (Beijing, China). Gram-negative *Escherichia coli* (*E. coli*, CMCC[B]44102), and Gram-positive *Staphylococcus aureus* (*S. aureus*, CMCC[B]26003) were the selected bacterial strains. White mushrooms were harvested in Dezhou, China. Prior to package testing, mushrooms with uniform characteristics (size, shape, and color) were selected as the experimental materials.

### 2.2. Starch Ultrasonication

The moisture and pH of the starch were 20% and 4.5–7.0 before ultrasonic treatment. The potato starch is composed of 80% large macromolecules of branched amylopectin and 20% amylose. The starch was sonicated (Frequency 20 kHz, Power 150 W) for 20 min with a VCX 750 ultrasonic processor (VCX 750, Sonics, Newtown, CT, USA) and then dried at 45 °C for 12h. They were grinded and sifted out to prepare the films.

### 2.3. Preparation of Nano-SiO_2_/Potato Starch Films

**Figure 1:**
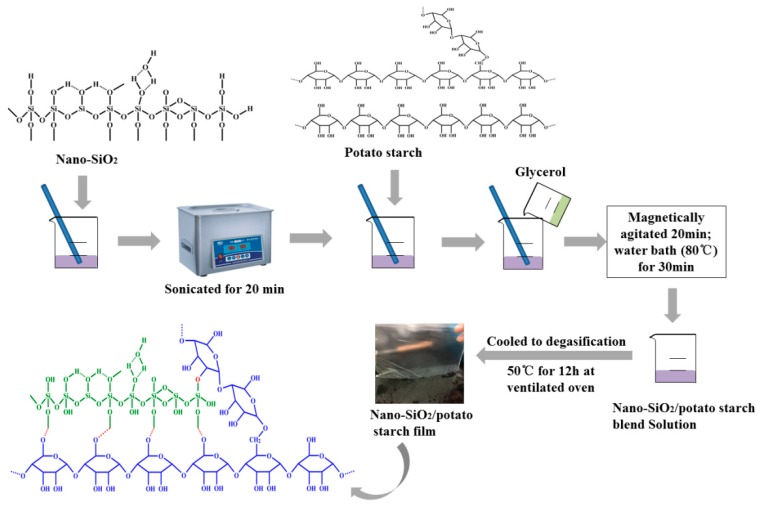
Schematic of the preparation of different films.

Pure potato starch films and potato starch/nano-SiO_2_ films were prepared by solvent casting. The process is illustrated in Figure 1. Prior to the preparation of nano-SiO_2_ solutions, 0.3% (*w*/*v* of water) nano-SiO_2_ of different sizes (15, 30, 80, and 100 nm) was added to 300 mL distilled water and then sonicated for 20 min to obtain a homogenous suspension. Subsequently, 5% (*w*/*v* of water) of potato starch and 5%(*w*/*v* of water) glycerin were added while the mixture was being shaken and gelatinized under 80 °C for 30 min The solution was allowed to cool down for degasification. Up to 100 mL of the solution was cast on a plastic utensil with a surface area measuring 25 cm × 25 cm and then dried in a vacuum oven at 50 °C. All uncovered samples were ultimately pretreated at 23 °C and 50% relative humidity (RH) in a humidity chamber for 24 h for the subsequent testing.

### 2.4. Physical Properties of the Films

#### 2.4.1. Water Vapor Transmission Rate

Water vapor transmission rate (WVTR) was measured gravimetrically in accordance with Chinese National Standards GB1037-88 (1998). The films were covered on the top of the weighing bottle containing 3 g anhydrous CaCl_2_ and then fixed with a rubber band. The weighing bottles were placed in a constant temperature and humidity chamber (25 °C RH 90%) for 24 h. WVTR was calculated as follows [23]:

(1) $$WVTR=\frac{m_{f}-m_{i}}{D\times S}$$

where *m*_f_ is the weight of the final bottle, and *m*_i_ is the weight of the initial bottle, *D* is the time, *S* is the effective area of the films, m^2^.

#### 2.4.2. Thickness, Oxygen Transmission Rate (OTR), Carbon Dioxide Transmission Rate (CDTR) and Water Resistance of the Films

The thickness of the films was measured using a hand-held micrometer with a precision of 0.01 mm as the mean of measurements obtained at 10 random points. The OTR and CDTR were measured according to Wang et al. (2014) [24]. The method described by Moreno et al. (2017) [25] was modified to measure the solubility and moisture absorption of the films. Their specific methods were described in the supplementary material.

### 2.5. Tensile Strength

The films were cut into rectangular strips (5 cm × 2 cm). A texture analyzer (TA-XT, Stable Micro Systems, UK) with the AMTG probe was used to determine the tensile strength of the films as an average of 6 measurements for each film. The initial distance of separation and speed were adjusted to 30 mm and 10 mm/s, respectively.

### 2.6. Optical Property

The transparency and light transmission of the films were measured using a ultraviolet visible spectrophotometer (UV-2550, Shimadzu International Trading Co., Ltd., Shanghai, China) by scanning the samples at wavelengths in the 200–800 nm range, with air as reference.

### 2.7. Thermal Properties

The thermal stability of the films was measured using a thermogravimetric analyzer (Netzsch STA449C, Selb, Germany). All films were scanned from 30 to 600 °C at a heating rate of 283.15 K/min under high-purity nitrogen. About 7 mg of each film was placed into a standard aluminum pan, with an empty pan used as the reference. Three replicates of each film were analyzed to ensure repeatability [26].

### 2.8. Characterization

#### 2.8.1. Scanning Electron Microscopy (SEM) of Native Starch and Films

The morphology of the films and starch were observed by SEM using an FEI Sirion 200 field-emission scanning electron microscope (FEI Sirion 200, FEI Co., Ltd., Hillsboro, OR, USA) operated at a voltage of 20 kV. Prior to observation, the starch and films were mounted on a cylindrical microscope stub covered with a carbon strip and sputter-coated with gold.

#### 2.8.2. Fourier Transform Infrared Spectroscopy (FTIR)

The chemical composition of the films was observed by FTIR (Nicolet 5700, Thermo Electron, Hampton, NH, USA) from 4000 to 600 cm^−1^ at a scanning rate of 4 cm^−1^. The samples were fixed directly in the sample holder and evaluated.

#### 2.8.3. X-ray Diffraction (XRD)

Diffraction patterns of the films were measured with an X-ray diffractometer (Bruker AXS Advance D8, Karlsruhe, Germany) coupled with Ni-filtered Cu and Kα radiation (accelerating voltage, 40 kV; current, 30 mA). The scattering angle range was set to 5°–45° following a scan rate of 1°/min.

### 2.9. Antibacterial Property

#### 2.9.1. Scanning Electron Microscope Assay

The bacterial cells were incubated in a Luria-Bertani (LB) medium (Peptone 10 g/L, Yeast extract 5 g/L, NaCl 10 g/L, Agar 20 g/L) at 37 °C overnight and then adjusted to 0.5–1 McFarland standards. The bacteria cells were then treated with nano-SiO_2_ suspension (0.3%, *w*/*v* of water) at different sizes for 2 h after the suspensions were centrifuged at 1500 g for 10 min. A 0.1 M phosphate buffer solution (PBS, pH 7.4) was used to wash the precipitated cells twice. Subsequently, 5% glutaraldehyde was employed to fix *E. coli* and *S. aureus* for 4 h under dark conditions. All samples were washed with PBS thrice and then dehydrated in various ethanol solutions. The treated *E. coli* and *S. aureus* were covered with gold by cathodic spraying. SEM (FEI Sirion 200, FEI Co., Ltd., Hillsboro, OR, USA) was ultimately used to observe the morphological changes in the bacterial cells.

#### 2.9.2. Agar Disk Diffusion Assay

The films were cut into a disc shape with a 6 mm diameter and then sterilized with 75% alcohol solution. They were preloaded on seeded agar plates, incubated at 37 °C for 24 h. Subsequently, the inhibitions against *E. coli* and *S. aureus* were measured as the diameters of the zones of inhibition around the films [27].

### 2.10. Preservation Property

White mushrooms (*A. bisporus*) were placed in 1050 mL polypropylene trays (14.0 cm × 8.50 cm × 6.80 cm). The polypropylene trays were tightly covered with nano-SiO_2_/potato starch films with nano-SiO_2_ of different sizes and then fixed with a rubber band. No-film packaging was used as the control. Each treatment was performed using 20 trays, with each tray containing about 160 g of white mushrooms. The weight loss, membrane permeability, hardness, surface color ∆*E*, and flesh color ∆*E* of white mushrooms were measured at intervals (0, 3, 6, 9, and 12 d) during storage at 4 °C.

Weight loss was determined by weighing the mushrooms before and during the storage period. The color (surface ∆*E* and flesh ∆*E*) was measured with a colorimeter (SC-80C, Kangguang Instrument Co., Ltd., Beijing, China). The hardness was measured with a GY-1 penetrometer (Mudanjiang Machinery Research Institute, Mudanjiang, China). The cell membrane permeability was expressed by tissue electrolyte leakage. Electrolyte leakage was measured following a procedure from Liu et al. (2010) [28]. The measurement was performed three times.

### 2.11. Statistical Analysis

The properties of the nano-SiO_2_/potato starch films with nano-SiO_2_ of different sizes were presented as means ± standard deviation at least in duplicates. All statistical analyses were performed using SPSS ver. 20 (SPSS Inc., Chicago, IL, USA) to analyze the variance. Duncan’s multiple-range test (*p* < 0.05) was employed to compare the differences.

## 3. Results and Discussion

### 3.1. SEM Analysis

**Figure 2:**
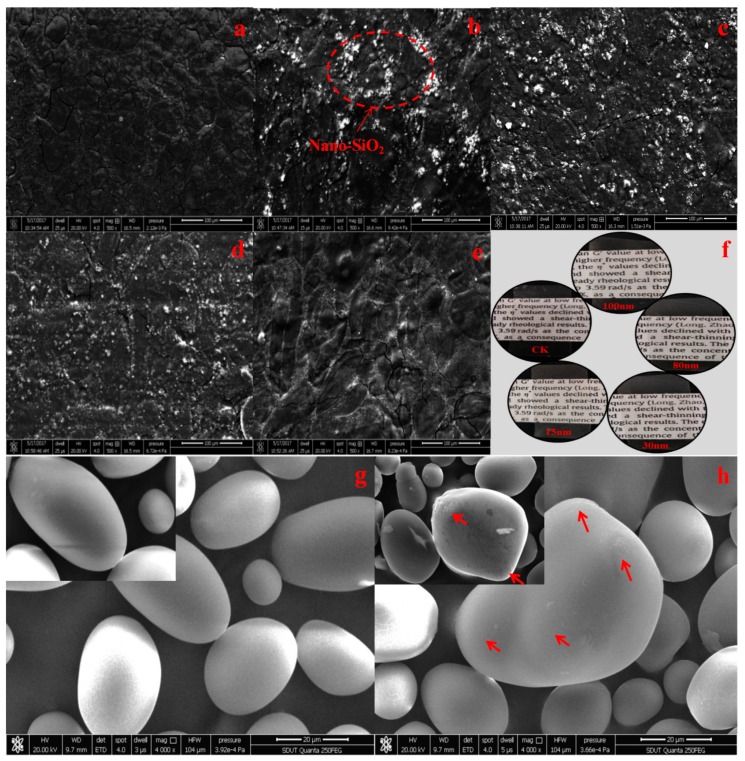
SEM and apparent images of starch and nano-SiO_2_/potato starch films. (**a**) CK: films without nano-SiO_2_; (**b**) films with the 15 nm nano-SiO_2_; (**c**) films with the 30 nm nano-SiO_2_; (**d**) films with the 80 nm nano-SiO_2_; (**e**) films with the 100 nm nano-SiO_2_; (**f**) the apparent images of different films (**g**) native potato starch; (**h**) potato starch after ultrasonication.

The surface morphology of films ultimately could reveal many properties of biodegradable materials. Figure 2 describes the microscopy of surface changes in the starch and nano-SiO_2_/potato starch films. The native potato starch was not uniform in shape and size, and the surface was flat and smooth. After ultrasonication, starch particles had rough surfaces with some spherical bulge, which formed by the aggregation of submicrocrystals and crystals. The dispersion of nanoparticle in the hosting matrix was associated with the macroscopic properties of the films [29]. Homogeneous dispersion of nanoparticles was necessary to successfully enhance the barrier properties of the films. As observed, the surface of the potato starch-based blend film without nano-SiO_2_ was smooth and homogenous, whereas that of the film with nano-SiO_2_ was rough. Moreover, 15 nm nano-SiO_2_ formed large aggregates, whereas those with increasing size showed less aggregation. The reason could be that the smaller particles had higher specific surface areas to form aggregates easily. This agglomeration could cause the large areas of the material to remain free from nanoparticles that influence the properties of the films. Therefore, the 100 nm nano-SiO_2_/potato starch films would exhibit the best barrier properties. This hypothesis will be discussed in a later section.

### 3.2. XRD Analysis

**Figure 3:**
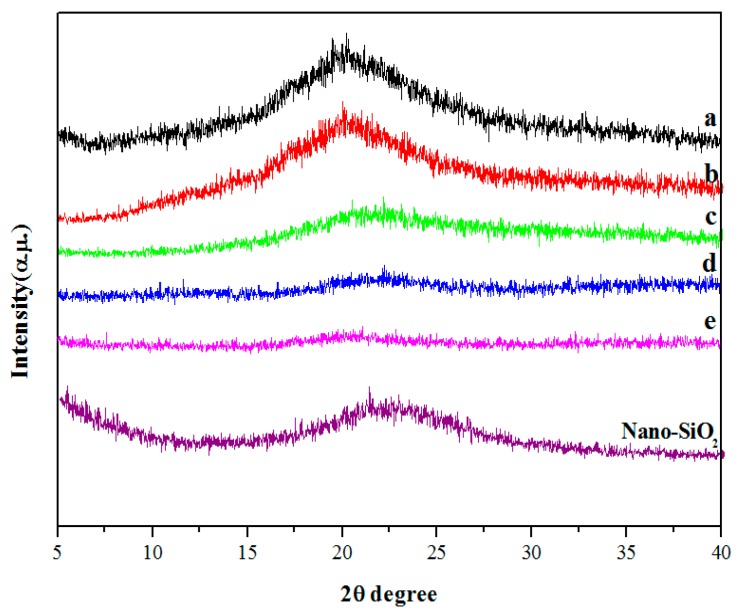
XRD patterns of the potato starch films with different size of nano-SiO_2_. (**a**) CK: films withoutnano-SiO_2_; (**b**) films with the 15 nm nano-SiO_2_; (**c**) films with the 30 nm nano-SiO_2_; (**d**) films with the 80 nm nano-SiO_2_; (**e**) films with the 100 nm nano-SiO_2_.

XRD can characterize the compatibility of films. When the crystalline and non-crystalline ingredients in the films exhibit good miscibility, the crystallinity becomes lower than that of the single-crystalline ingredient [30]. The XRD of nano-SiO_2_/potato starch films was presented in Figure 3. The characteristic diffraction peak of the potato starch films without nano-SiO_2_ appeared clearly at around 20°, which could be attributed to the crystalline and amorphous fractions of the potato starch [31]. Comparison of the patterns with the addition of nano-SiO_2_ indicated that the diffraction peak weakened with an increase in size. The intermolecular interactions between potato starch and nano-SiO_2_ led to the dispersion of the nano-SiO_2_ molecules into the potato starch matrix. Meanwhile, nano-SiO_2_ destroyed the original crystalline domains of the potato starch films to decrease the crystallinity. In addition, nano-SiO_2_ of a smaller particle size and a larger surface was easier to reunite. Thus, the 100 nm nano-SiO_2_ could be more easily dispersed in the potato starch films and disrupt the regularity of the arrangement among the potato starch and nano-SiO_2_ molecules, thereby decreasing the crystallinity of nano-SiO_2_/potato starch films. The results supported the conclusion drawn from SEM that potato starch and nano-SiO_2_ exhibited good compatibility and that optimal dispersibility was obtained with the 100 nm nano-SiO_2_.

### 3.3. FTIR Analysis

**Figure 4:**
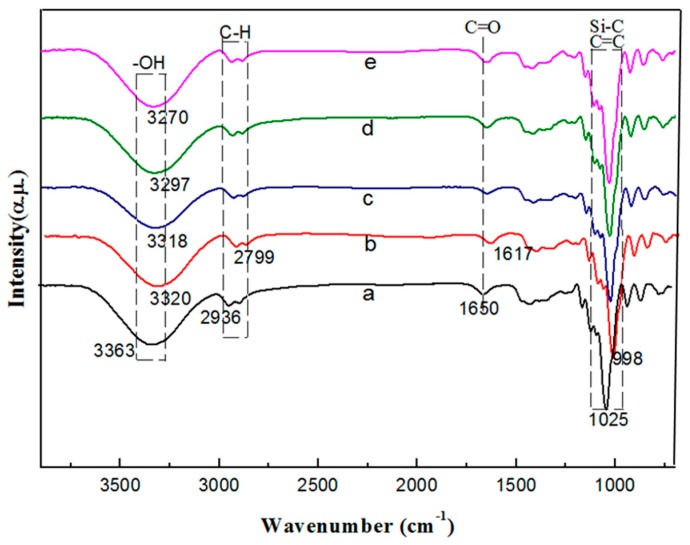
FTIR of the films with nano-SiO_2_ of different sizes. (**a**) CK: films withoutnano-SiO_2_; (**b**) films with the 15 nm nano-SiO_2_; (**c**) films with the 30 nm nano-SiO_2_; (**d**) films with the 80 nm nano-SiO_2_; (**e**) films with the 100 nm nano-SiO_2_.

FTIR spectroscopy as a powerful technique can determine the miscibility of polymers. The detailed chemical bond structures of the films at 4000–400 cm^−1^ were presented in Figure 4. A strong and broad adsorption band appeared at 3340 cm^−1^ because of the –OH groups on the potato starch backbone. The H–O–H stretching vibration absorption appeared at 1650 cm^−1^, and the peak at 2936 cm^−1^ was ascribed to the C–H stretching vibration [32]. The peaks at about 928, 992, and 1025 cm^−1^ represented the C–O–C stretching vibration in the starch structure. With the nano-SiO_2_ of different sizes added, the strong absorption band widened at 3340 cm^−1^ and was then transferred to the low wavenumbers. The 100 nm nano-SiO_2_ became dominant and shifted to 3335 cm^−1^. In addition, the peak at 2936 cm^−1^ moved to 2933 cm^−1^. The peaks at 1025 cm^−1^ were also transferred to the low wavenumbers, indicating that the active group of potato starch formed the hydrogen bond with –OH on the nano-SiO_2_ surface. The peaks at 1260 and 987 cm^−1^ were attributed to the Si–C stretching vibration and bending vibration [33], the peaks at 1072 and 922 cm^−1^ were attributed to the Si–O stretching vibration and Si–H bending vibration. It indicated that the intermolecular hydrogen bonding between nano-SiO_2_ and potato starch increased. The FTIR results confirmed that the compatibility between nano-SiO_2_ and potato starch was attributed to intermolecular hydrogen bonding, and the 100 nm nano-SiO_2_ could be dispersed most uniformly during film formation and blending [22]. The finding was consistent with the SEM and XRD results, indicating that the 100 nm nano-SiO_2_ exhibited good miscibility.

### 3.4. TG and DTG Analysis

**Figure 5:**
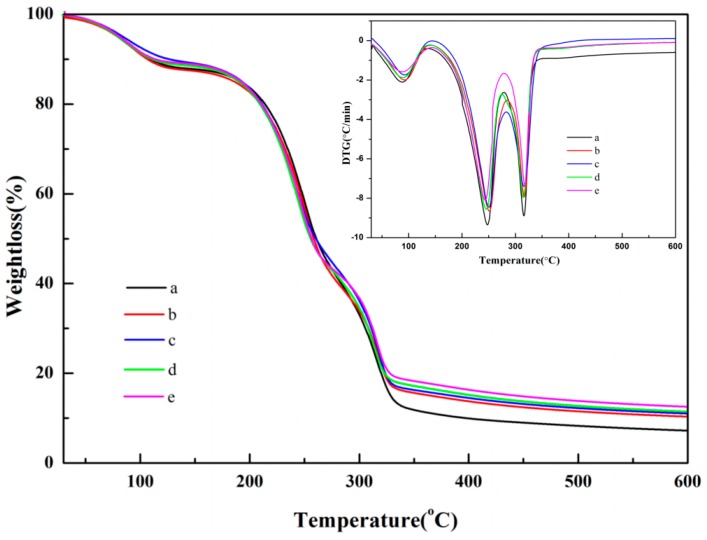
TG and DTG curves of the films. (**a**) CK: films withoutnano-SiO_2_; (**b**) films with the 15 nm nano-SiO_2_; (**c**) films with the 30 nm nano-SiO_2_; (**d**) films with the 80 nm nano-SiO_2_; (**e**) films with the 100 nm nano-SiO_2_.

The thermal stability of the films was determined by TG and DTG. The weight loss thermograms of the films were shown in Figure 5. Three well-defined stages represent the weight loss of all films in the following ranges: (i) 100–200 °C, (ii) 200–330 °C, and (iii) 330–600 °C. The first stage showed a small weight loss (about 10%) that was attributed to moisture from the surface or interior of the films. The decomposition rate of the nano-SiO_2_/potato starch films was lower than that of the potato starch films. This difference indicated that the motion and reaction of water molecules were hindered with the addition of nano-SiO_2_ [34]. The second stage demonstrated the thermal expansion of films with a weight loss of 60%–70%. This occurrence was attributed to the loss of the adjacent hydroxyl group and cleavage of the glycosidic bond [9]. Compared with that of the pure potato starch films, the weight loss decreased in the range of 270–330 °C with the addition of nano-SiO_2_ because of the strong interaction between the nano-SiO_2_ and potato starch. The potato starch films had a weight loss of 70.1% from 200 to 330 °C, whereas the nano-SiO_2_/potato starch films (15, 30, 80, and 100) had weight losses of 67.4%, 66.0%, 65.4%, and 60.5%, respectively, at the same temperature. These losses demonstrated that strong interactions between starch molecules and Si–OH in nano-SiO_2_ delayed the movement of the molecular chains to strengthen the thermostability of the films [35,36]. These results were consistent with the results of FTIR. The third stage showed the weight loss was in ceases, indicating that such behavior was related to the crystallinity of the films when dissolved nano-SiO_2_ was dispersed in the matrix of the potato starch films. The result was identified in the XRD pattern. The amount of residue in the films with nano-SiO_2_ of different sizes followed the order 100 nm > 80 nm > 30 nm > 15 nm. The results indicated that the 100 nm nano-SiO_2_/potato starch films exhibited the highest thermostability because nano-SiO_2_ was more homogeneous in the films.

### 3.5. Physical and Mechanical Properties of the Films

#### 3.5.1. Water Vapor Transmission Rate Analysis

**Figure 6:**
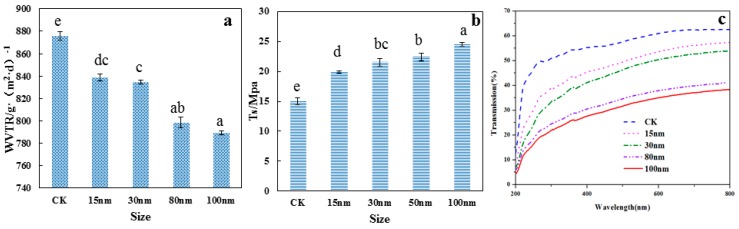
The WVTR (**a**), *T*_s_ (**b**), and transparency (**c**) of the potato starch films with nano-SiO_2_ of different sizes.

The WVTR could represent moisture transport through films. This measurement was a key factor for maintaining the good quality of food against water adsorption and desorption in food packaging [37]. The WVTRs of nano-SiO_2_/potato starch films with different sizes are given in Figure 6a. The WVTRs of the films decreased because of the increase in nano-SiO_2_ size. The SEM image (Figure 1) of the films showed compact structures when nano-SiO_2_ particles with different sizes were added. The phenomenon was ascribed to the hydrogen bonds between the oxygen atoms of nano-SiO_2_ and potato starch matrix. In addition, the good dispersion of nano-SiO_2_ in the potato starch matrix provided curving paths for water molecules to across the films [38]. In a previous study, the WVTR was also affected by the degree of crystallinity, size of the nano-particles, free volume, and macro-voids [39]. The 100 nm nano-SiO_2_/potato starch films obtained the lowest WVTR (789.41 g/m^2^⋅d), which was ascribed to the decrease in crystallinity of the 100 nm nano-SiO_2_ to a greater extent (Figure 2). The small size of nano-SiO_2_ was found to facilitate the dispersion in the films, resulting in a low WVTR [40].

#### 3.5.2. Tensile Strength Analysis

The tensile strengths (*T*_s_) of the films with nano-SiO_2_ of different sizes were given in Figure 6b. The control potato starch films exhibited a lower tensile strength (15.1 MPa) than that of the nano-SiO_2_/potato starch films. The incorporation of the nano-SiO_2_ significantly affected the tensile strength of the potato starch films. The matrix of the potato starch films could be strengthened by O–Si–O bonding, hydrogen bonding, or electrostatic attraction with the incorporation of nano-SiO_2_ [41]. The optimal tensile strength was obtained when the 100 nm nano-SiO_2_ was used. The 100 nm nano-SiO_2_ was well adsorbed and bonded with potato starch in the formation of the films, thus improving the interphase adhesion strength between the matrix and the nano-SiO_2_. In addition, the smaller nano-SiO_2_ was more prone to agglomeration in the dispersion, indicating that their high surface energy led to the aggregation of nano-SiO_2_ and destroyed the original integrity of the films [37]. This finding agreed with the SEM results. This reinforcing effect of nano-SiO_2_ was consistent with the previous study by Jiang et al. (2016) [35].

#### 3.5.3. Optical Property Analysis

The spectroscopic images of the films with nano-SiO_2_ of different sizes from 200 to 800 nm were presented in Figure 6c. The transmittance of the films gradually increased as the wavelength increased. The addition of nano-SiO_2_ caused low transmittance, indicating that the nano-SiO_2_/potato starch films could resist against UV light, restraining the deterioration of food caused by UV radiation [42]. Notably, the UV light barrier values presented in this study for the nano-SiO_2_/potato starch films prepared using 100 nm nano-SiO_2_ were higher than those other films. Only 30% UV light could pass through the 100 nm films at 600 nm. The result indicated that the 100 nm nano-SiO_2_ could be uniformly distributed in the potato starch matrix. The size of nano-SiO_2_ could affect the transmittance of the films, which was consistent with the findings of Hassannia-Kolaee et al. (2016) [23].

#### 3.5.4. Thickness

The thickness of the films with nano-SiO_2_ of different sizes is presented in Figure S1a (Supplementary Materials). The thicknesses of the films ranged from 0.087 to 0.091mm. No significant difference was observed between the different films (*p* > 0.05). Many studies reported that film thickness could affect the properties of the films [43]. This finding showed that the thicknesses of the films with nano-SiO_2_ of different sizes in the present study showed no significant difference to accurately investigate their properties.

#### 3.5.5. CDTR and OTR Analysis

Many degradation reactions in foods occur because of oxygen, which changes the nutritional and sensory value of food, shortening their shelf life. Oxidation mainly led to these reactions, such as vitamins loss, enzymatic browning, and fat rancidity. Therefore, the functions of packaging materials for reducing the deteriorative effects of oxygen were of great concern [44]. The starch films had the advantage of a lower OTR than that of synthetic films [45]. Figure S1b,c (Supplementary Materials) showed that the gas transmission rate (OTR and CDTR) decrease with the addition of nano-SiO_2_. During packaging, O_2_ and CO_2_ would first dissolve on the high-pressure side of the films. The difference in pressure could cause the gas molecules to be stuck in the torque hole, leading to a violent movement of the macromolecular chains of the films. O_2_ and CO_2_ would gradually penetrate along this channel and finally be released from the side with low partial pressure [8]. This occurrence indicated that dispersed nano-SiO_2_ changed the micropore structure of the potato starch films and that the Si–O–Si groups in the films could control the exchange of O_2_ and CO_2_. Meanwhile, OTR and CDTR decreased with an increase in nano-SiO_2_ size. The OTR and CDTR of the 100 nm films were 111.54 g/m^2^⋅d and 902.47 g/m^2^⋅d, respectively, which could inhibit the respiration of fruits and vegetables in the package. The reason might be that the nano-SiO_2_ particles with a smaller size and a larger surface area were severely agglomerated to reduce hydrogen bonds, leading to the increased gas permeabilities of the films. Sun et al. also reported that the gas permeabilities decreased, which was caused by the dispersibility of the nano-SiO_2_.

#### 3.5.6. Water Resistance

The effect of nano-SiO_2_ size on the moisture absorption and solubility of the films is shown in Figure S1d,e (Supplementary Materials). The moisture absorption and solubility of the films were markedly decreased with an increase in size of the nano-SiO_2_. The intermolecular hydrogen bonds between nano-SiO_2_ and potato starch increased the cohesiveness of the film matrix by the formation of a network structure. Therefore, nano-SiO_2_ enhanced the moisture absorption and solubility of the films [40,46]. Furthermore, the100nm nano-SiO_2_ was uniformly distributed in the potato starch matrix, resulting in a good interaction between the nano-SiO_2_ and the potato starch. This effect indicated that the total void volume of the water molecules was reduced in the microstructures of the films. This result was consistent with SEM and FTIR. Consequently, the nano-SiO_2_/potato starch films (100 nm) exhibited the optimal barrier and water resistance properties, which the moisture absorption (18.4%) and solubility (52.7%) were lower than other films.

### 3.6. Antibacterial Property Analysis

#### 3.6.1. Scanning Electron Microscopy

**Figure 7:**
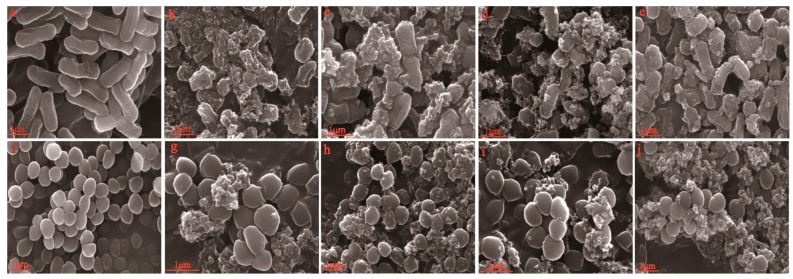
SEM of *E. coli* and *S. aureus*. (**a**) Image of untreated *E. coli*; (**b**), (**c**), (**d**), and (**e**) Images of *E. coli* treated with nano-SiO_2_ particles measuring 5, 30, 80, and 100 nm; (**f**) Image of untreated *S. aureus*; (**g**), (**h**), (**i**), and (**j**) Images of *E. coli* and *S. aureus* treated with nano-SiO_2_ particles measuring 15, 30, 80, and 100 nm.

Figure 7 described the SEM images of *E. coli* and *S. aureus* treated with nano-SiO_2_ of different sizes. The images illustrate the antibacterial activity of nano-SiO_2_ on the tested bacteria. Compared with those of the untreated controls, the morphological surfaces of the treated bacteria were markedly changed. The untreated *E. coli* appeared regular, rod-shaped, and intact (Figure 7a). The untreated *S. aureus* appeared intact and spherical (Figure 7f). Some of the *E. coli* treated with nano-SiO_2_ became deformed, shriveled, pitted, adhered to each other, and parts of the cell were broken, which could lead to the leaching out of genetic materials and nutrients [35]. Moreover, the changes were more severe and evident with a decrease in nano-SiO_2_ size (Figure 7b–e), indicating that the 15 nm nano-SiO_2_ showed an extremely porous structure and high surface activity following the sufficient adsorption properties [13]. Moreover, the boundaries of the bacteria were irregular and wrinkled. However, nano-SiO_2_ was less efficient against *S. aureus* (Figure 7g–j). The difference could be explained by the structural difference in *E. coli* and *S. aureus*. Both bacteria exhibited resemblance in their internal structures, but now also in their external structures. The cells of *E. coli* had an outer membrane composed of proteins, phospholipids, and lipopolysaccharide, possessing a thin peptidoglycan layer. The cell of *S. aureus* possessed a thick peptidoglycan layer containing lipoteichoic acids and teichoic. Thus, higher concentrations of nano-SiO_2_ and longer contact time for *S. aureus* would be required to obtain the same effect as that of *E. coli* [13]. Relevant investigations should be further conducted to clarify its mechanism.

#### 3.6.2. Antibacterial Activity of the Films

**Figure 8:**
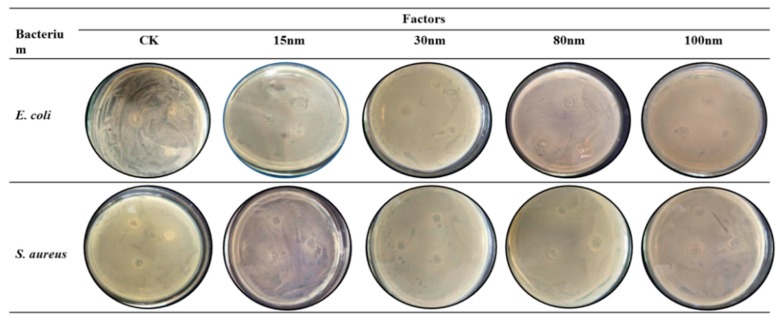
Inhibitory effect of potato starch films with nano-SiO_2_ of different sizes against *E. coli* and *S. aureus*.

The films with nano-SiO_2_ of different sizes for their antibacterial activity against *E. coli* and *S. aureus* are presented in Figure 8 and Table S1 (Supplementary Materials). The inhibitory effect was determined based on the clear zone around the film discs. The zones of inhibition around the films included the diameter of the film discs. This finding suggested the absence of antibacterial activity because no clear zone was observed around the films. All the nano-SiO_2_/potato starch films were not infested with bacteria, indicating that external bacteria were prevented from passing through the films to infect food in the packaging process. The nano-SiO_2_/potato starch films showed a significant inhibitory effect against *E. coli* and low efficiency against *S. aureus*. The nano-SiO_2_ could be adsorbed on the cell walls of the bacteria, destroying the structure of the cell membranes. The structure of the cell organelles was then destroyed because of the permeation of the nano-SiO_2_. Thus, nano-SiO_2_ could damage the antioxidant system and produce stronger lipid peroxidation. In addition, the inhibitory zone of the films with 15 nm nano-SiO_2_ measured 1.2 cm. However, part of the films with 15 nm nano-SiO_2_ had no inhibitory zone because of the aggregation of 15 nm nano-SiO_2_ in the potato starch films. The inhibitory zone of the films with 100 nm nano-SiO_2_ measured 0.8 cm, and all samples had inhibitory zones. This finding indicated that the films with 100 nm nano-SiO_2_ exhibited a higher antibacterial activity. Therefore, the uniform dispersion of the nano-SiO_2_ with small particles in the potato starch films plays a crucial role in fruit and vegetable preservation.

### 3.7. Preservation Properties of the Films on White Mushrooms (A. Bisporus)

**Figure 9:**
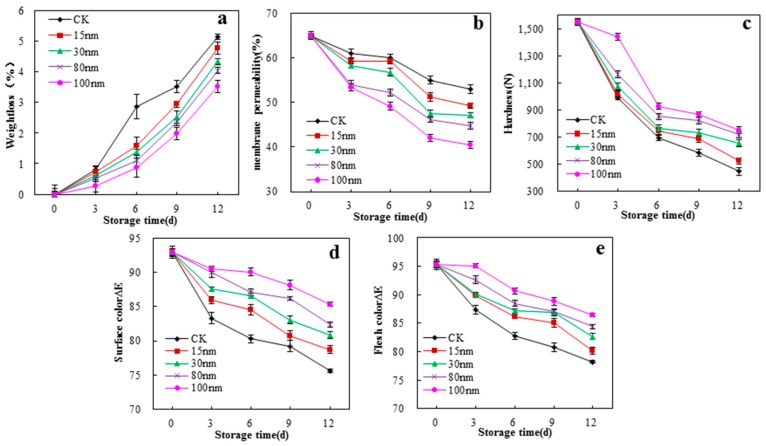
Effects of the films on weight loss (**a**), membrane permeability (**b**), hardness (**c**), surface ∆*E* (**d**), and flesh ∆*E* (**e**) of white mushroom during storage at 4 ± 1 °C.

To determine the change in the quality of white mushrooms packaged in nano-SiO_2_/ potato starch films during storage, the weight loss, membrane permeability, hardness, and color were evaluated (Figure 9). After the postharvest of white mushrooms, the weight loss, membrane permeability, hardness, and color deteriorated because of high metabolic activity, respiration rate, and dehydration. The results indicated that membrane permeability, hardness, and color of white mushrooms decreased, and weight loss increased with storage. Meanwhile, all quality indices changed slowly with the potato starch/nano-SiO_2_ films. Moreover, the nano-SiO_2_ /potato starch films exhibited better storage and preservation properties (Figure S1, Supplementary Materials) as the size of the nano-SiO_2_ increased. The 100 nm nano-SiO_2_ was uniformly dispersed in the potato starch films and combined with potato starch by hydrogen and chemical bonding. Therefore, the 100 nm nano-SiO_2_/potato starch films in the package could reduce the permeation of gas and water, as well as inhibit the growth of microorganism, strengthen the mechanical properties, and improve the microstructures of the films. This enhancement delayed the change in the quality of white mushrooms within the storage period.

## 4. Conclusions

This study demonstrated that the size of nano-SiO_2_ played an important role in the physical and mechanical properties of potato starch films. The 100 nm nano-SiO_2_ was dispersed more uniformly in the potato starch films and the strong hydrogen bond was formed between the nano-SiO_2_ and potato starch molecules. The packing properties of the 100 nm nano-SiO_2_/potato starch films were superior to those of the other films, which the WVTR, OTR, CDTR, solubility, moisture absorption, and tensile strength were 789.41 g/m^2^⋅d, 111.54 g/m^2^⋅d, 902.47 g/m^2^⋅d, 52.7%, 18.4% and 15.1MPa, respectively. Especially, the resistance to ultraviolet aging and thermal aging was enhanced by the incorporation of nano-SiO_2_. The antibacterial experiment indicated that the nano-SiO_2_/potato starch films exhibited good antibacterial activity against the *E. coli* and was less efficient against the *S. aureus*. The effects of the preservation properties of the nano-SiO_2_/potato starch films on the white mushrooms were remarkable; and those of the 100 nm films were superior. Further investigations should be conducted to the dispersion of nano-SiO_2_ with a small size in the potato starch films. The mechanism underlying the antibacterial activity of nano-SiO_2_/potato starch films against microorganism, particularly those responsible for the pathogenic bacteria of postharvest white mushroom during storage, also needs to be expounded.

## Supplementary Materials

The following are available online at http://www.mdpi.com/2073-4360/10/10/1172/s1, Figure S1: Thickness (a), OTR (b), CDTR (c), solubility (d), and moisture absorption (e) of the potato starch films with nano-SiO_2_ of different sizes. Table S1: Inhibition zone diameter of potato starch films with different size of nano-SiO_2_ against *E. coli* and *S. aureus*.

## References

[1] Vargas C.G., Costa T.M.H., Rios A.D.O., Flôres S.H. (2017). Comparative study on the properties of films based on red rice (oryza glaberrima) flour and starch. Food Hydrocoll..

[2] Pagno C.H., Costa T.M.H., De Menezes E.W., Benvenutti E.V., Hertz P.F., Matte C.R., Tosati J.V., Monteiro A.R., Rios A.O., Flôres S.H. (2015). Development of active biofilms of quinoa (*Chenopodium quinoa* W.) starch containing gold nanoparticles and evaluation of antimicrobial activity. Food Chem..

[3] Piñeros-Hernandez D., Medina-Jaramillo C., López-Córdoba A., Goyanes S. (2017). Edible cassava starch films carrying rosemary antioxidant extracts for potential use as active food packaging. Food Hydrocoll..

[4] Wang W.T., Zhang H., Jia R., Dai Y.Y., Dong H.Z., Hou H.X., Guo Q.B. (2018). High performance extrusion blown starch/polyvinyl alcohol/clay nanocomposite films. Food Hydrocoll..

[5] Podshivalov A., Zakharova M., Glazacheva E., Uspenskaya M. (2017). Gelatin/potato starch edible biocomposite films: correlation between morphology and physical properties. Carbohydr. Polym..

[6] Oleyaei S.A., Almasi H., Ghanbarzadeh B., Moayedi A.A. (2016). Synergistic reinforcing effect of TiO_2_ and montmorillonite on potato starch nanocomposite films: Thermal, mechanical and barrier properties. Carbohydr. Polym..

[7] Ortega F., Giannuzzi L., Arce V.B., García M.A. (2017). Active composite starch films containing green synthetized silver nanoparticles. Food Hydrocoll..

[8] Sun T., Wu C.L., Hao H., Dai Y., Li J.R. (2016). Preparation and preservation properties of the chitosan coatings modified with the in situ, synthesized nano siox. Food Hydrocoll..

[9] Yang M.L., Shi J.S., Xia Y.Z. (2018). Effect of SiO_2_, PVA and glycerol concentrations on chemical and mechanical properties of alginate-based films. Int. J. Biol. Macromol..

[10] Tang H., Xiong H., Tang S., Peng Z. (2009). A starch-based biodegradable film modified by nano silicon dioxide. J. Appl. Polym. Sci..

[11] Torabi Z., Mohammadinafchi A. (2013). The effects of SiO_2_ nanoparticles on mechanical and physicochemical properties of potato starch films. J. Chem. Health Risks.

[12] Rahman P.M., Mujeeb V.M.A., Muraleedharan K., Thomas S.K. (2018). Chitosan/nano-ZnO composite films: Enhanced mechanical, antimicrobial and dielectric properties. Arabian J. Chem..

[13] Talebian N., Zare E. (2014). Structure and antibacterial property of nano-SiO_2_ supported oxide ceramic. Ceram. Int..

[14] Song H.W., Yuan W.M., Jin P., Wang W., Wang X.F., Yang L.M., Zhang Y.F. (2016). Effects of chitosan/nano-silica on postharvest quality and antioxidant capacity of loquat fruit during cold storage. Postharvest Biol. Technol..

[15] Shi S.Y., Wang W., Liu L.Q., Wu S.J., Wei Y.Z., Li W.C. (2013). Effect of chitosan/nano-silica coating on the physicochemical characteristics of longan fruit under ambient temperature. J. Food Eng..

[16] Tabatabaei R.H., Jafari S.M., Mirzaei H., Nafchi A.M., Dehnad D. (2018). Preparation and characterization of nano-SiO_2_ reinforced gelatin-kcarrageenan biocomposites. Int. J. Biol. Macromol..

[17] Liu X., Cui Y.H., Chen H.Y. (2018). Influence of depositing nano-SiO_2_ particles on the surface microstructure and properties of jute fibers via in situ synthesis. Compos. Part A.

[18] Ritamäki M., Rytöluoto I., Lahti K., Karttunen M. Effects of thermal aging on the characteristic breakdown behavior of Nano-SiO_2_-BOPP and BOPP films. Proceedings of the 11th International Conference on the Properties and Applications of Dielectric Materials (ICPADM).

[19] Gong C., Yang L.Q., Zhou J.C., Xiang G., Zhuang Z.X. (2017). Possible role of PAPR-1 in protecting human HaCaT cells against cytotoxicity of SiO_2_ nanoparticles. Toxicol. Lett..

[20] Zhao F.C., Zeng X.R., Li H.Q., Zhang J. (2012). Preparation and characterization of nano-SiO_2_/fluorinated polyacrylate composite latex via nano-SiO_2_/acrylate dispersion. Colloids Surf. A.

[21] Cui X., Zhong S., Yan J., Wang C., Zhang H., Wang H. (2010). Synthesis and characterization of core–shell SiO_2_-fluorinated polyacrylate nanocomposite latex particles containing fluorine in the shell. Colloids Surf. A.

[22] Fortuni B., Fujita Y., Ricci M., Inose T., Aubert R., Lu G. (2017). A novel method for in situ synthesis of sers-active gold nanostars on polydimethylsiloxane film. Chem. Commun..

[23] Hassannia-Kolaee M., Khodaiyan F., Pourahmad R., Shahabi-Ghahfarrokhi I. (2016). Development of ecofriendly bionanocomposite: Whey protein isolate/pullulan films with nano-SiO_2_. Int. J. Biol. Macromol..

[24] Wang L., Xiao M., Dai S.H., Song J., Ni X.W., Fang Y.P., Corke H., Jiang F.T. (2014). Interactions between carboxymethyl konjac glucomannan and soy protein isolate in blended films. Carbohydr. Polym..

[25] Moreno O., Cárdenas J., Atarés L., Chiralt A. (2017). Influence of starch oxidation on the functionality of starch-gelatin based active films. Carbohydr. Polym..

[26] Colussi R., Pinto V.Z., Slm E.H., Biduski B., Prietto L., Castilhos D.D., Zavareze E.D.R., Dias A.R.G. (2016). Acetylated rice starches films with different levels of amylose: Mechanical, water vapor barrier, thermal, and biodegradability properties. Food Chem..

[27] Vimala K., Yallapu M.M., Varaprasad K., Reddy N.N., Ravindra S., Naidu N.S., Raju K.M. (2011). Fabrication of curcumin encapsulated chitosan–PVA silver nanocom posite films for improved antimicrobial activity. J. Biomater. Nanobiotechnol..

[28] Liu Z.L., Wang X.Y., Zhu J.Y., Wang J. (2010). Effect of high oxygen modified atmosphere on post-harvest physiology and sensorial qualities of mushroom. Int. J. Food Sci. Technol..

[29] Lizundia E., Vilas J.L., Sangroniz A., Etxeberria A. (2017). Light and gas barrier properties of PLLA/metallic nanoparticles composite films. Eur. Polym. J..

[30] Wu C.H., Peng S.H., Wen C.R., Wang X.M., Fan L.L., Deng R.H., Pang J. (2012). Structural characterization and properties of konjac glucomannan/curdlan blend films. Carbohydr. Polym..

[31] Sahraee S., Milani J.M., Ghanbarzadeh B., Hamishehkar H. (2017). Physicochemical and antifungal properties of bio-nanocomposite film based on gelatin-chitin nanoparticles. Int. J. Biol. Macromol..

[32] Ghosh Dastidar T., Netravali A. (2013). Cross-linked waxy maize starch-based green composites. ACS Sustain. Chem. Eng..

[33] Yong C., Xie K., Pan Y., Chen Y., Xu J., Xing X. (2000). Synthesis of Si/C/N ultrafine composite powder by pyrolysis of polynirisilicane. J. Chin. Ceram. Soc..

[34] Zhao Y.L., Qi X.W., Dong Y., Ma J., Zhang Q.L., Song L.Z., Yang Y.L., Yang Q.X. (2016). Mechanical, thermal and tribological properties of polyimide/nano-SiO_2_ composites synthesized using an in-situ polymerization. Tribol. Int..

[35] Jiang S.S., Liu C.Z., Wang X.J., Xiong L., Sun Q.J. (2016). Physicochemical properties of starch nanocomposite films enhanced by self-assembled potato starch nanoparticles. LWT-Food Sci. Technol..

[36] Dai L., Qiu C., Xiong L., Sun Q. (2015). Characterisation of corn starch-based films reinforced with taro starch nanoparticles. Food Chem..

[37] Shahabi-Ghahfarrokhi I., Khodaiyan F., Mousavi M., Yousefi H. (2015). Preparation of UV-protective kefiran/Nano-ZnO nanocomposite: physical and mechanical properties. Int. J. Biol. Macromol..

[38] Kristo E., Biliaderis C.G. (2007). Physical properties of starch nanocrystal-reinforced pullulan films. Carbohyd. Polym..

[39] Almasi H., Ghanbarzadeh B., Entezami A.A. (2010). Physicochemical properties of starch–CMC–nanoclay biodegradable films. Int. J. Biol. Macromol..

[40] Xiong H.G., Tang S.W., Tang H.L., Zou P. (2008). The structure and properties of a starch-based biodegradable film. Carbohydr. Polym..

[41] Farhan A., Hani N.M. (2017). Characterization of edible packaging films based on semi-refined kappa-carrageenan plasticized with glycerol and sorbitol. Food Hydrocoll..

[42] Luchese C.L., Garrido T., Spada J.C., Tessaro I.C., La Caba K.D. (2018). Development and characterization of cassava starch films incorporated with blueberry pomace. Int. J. Biol. Macromol..

[43] Biduski B., Silva F.T., Silva W.M., Halal S.L., Pinto V.Z., Dias A.R. (2017). Impact of acid and oxidative modifications, single or dual, of sorghum starch on biodegradable films. Food Chem..

[44] Guerrero P., Hanani Z.A.N., Kerry J.P., Caba K.D.L. (2011). Characterization of soy protein-based films prepared with acids and oils by compression. J. Food Eng..

[45] Nawab A., Alam F., Haq M.A., Lutfi Z., Hasnain A. (2017). Mango kernel starch-gum composite films: Physical, mechanical and barrier properties. Int. J. Biol. Macromol..

[46] Zou W., Peng J., Yang Y., Zhang L., Liao B., Xiao F. (2007). Effect of nano-SiO_2_, on the performance of POLY (MMA/BA/MAA)/EP. Mater. Lett..

