# Supplementary material for: Preparation and Characterization of Potato Starch Film with Various Size of Nano-SiO2
Source: Polymers (Basel). 2018 Oct 22;10(10):1172. doi: 10.3390/polym10101172 (PMC6403978; doi:10.3390/polym10101172)
Supplement: Supplementary file 1 [file polymers-10-01172-s001.pdf]

Supplementary Materials: Preparation and Characterization of Potato Starch Film with Various Size of Nano-SiO_2

Rongfei Zhang, Xiangyou Wang and Meng Cheng

1. Thickness, oxygen transmission rate (OTR), carbon dioxide transmission rate (CDTR) and water resistance of the films

The thickness of the films was measured using a hand-held micrometer with a precision of 0.01 mm as the mean of measurements obtained at 10 random points.

The oxygen transmission rate (OTR) and carbon dioxide transmission rate (CDTR) of the films were measured by chemical absorption. About 3–5 g of the deoxidizer/KOH was placed in a weighing bottle. The bottle was covered with the different films and then fixed with a rubber band. They were then placed in a constant temperature and humidity chamber (23 °C, RH 90%) for 48 h. The increase in weight was measured until it remained constant. O_2/CO_2 was absorbed by the deoxidizer /KOH through the films. OTR and CDTR were calculated as follows (Wu et al., 2012; Wang et al., 2014):

$$\mathrm{OTR} = \frac{\Delta m_1}{\mathrm{D} \times \mathrm{S}} \tag{1}$$

$$\mathrm{CDTR} = \frac{\Delta \mathrm{m}_2}{\mathrm{D} \times \mathrm{S}} \tag{2}$$

where Δm_1 is the amount of O_2 absorbed by the deoxidizer, Δm_2 is the amount of CO_2 absorbed by KOH, D is the storage time, and S is the effective area of the films.

The method described by Moreno et al. (2017) was modified to measure the solubility and moisture absorption of the films. The solubility of the films was calculated according to Equation (3):

$$\mathrm{Solubility}(\%) = \frac{\mathrm{m}_0 - \mathrm{m}_1}{\mathrm{m}_0} \tag{3}$$

where m_0 is the initial weight before immersion; m1 is the weight of undissolved dry samples.

Moisture absorption was calculated as follows:

$$\text{Moisture absorption } (\%) = \frac{\mathrm{m}_2 - \mathrm{m}_o'}{\mathrm{m}_2} \tag{4}$$

where $m_0'$is the initial weight of films before treatment; m_2 is the weight of films after placed in desiccators containing a saturated calcium-nitrite solution at 25 °C to ensure an RH of 60% for 6h.

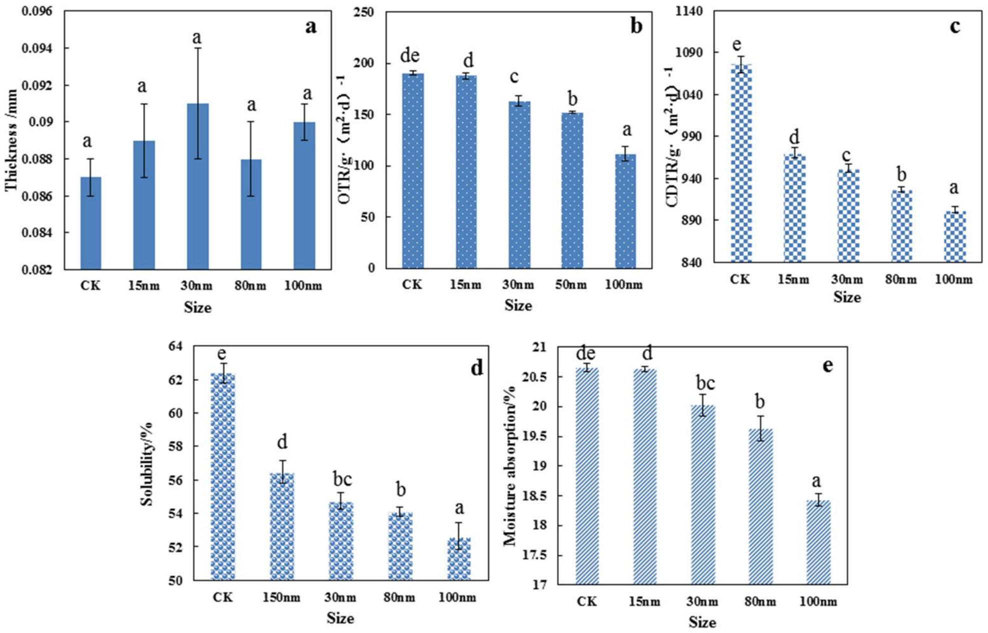

Figure S1. Thickness (a), OTR (b), CDTR (c), solubility (d), and moisture absorption (e) of the potato starch films with nano-SiO_2 of different sizes.

Table S1. Inhibition zone diameter of potato starch films with different size of nano-SiO_2 against *E. coli* and *S. aureus.*

Indicator organisms	Inhibition zone diameter (mm)				
	CK	15 nm	30 nm	80 nm	100 nm
	6.0	12.0 ± 0.10 ^{a}	8.6 ± 0.09 ^{a}	8.3 ± 0.05 ^{a}	8.0 ± 0.08 ^{a}
E. coli	6.0	8.1 ± 0.04 ^{b}	6.0 ± 0.02 ^{b}	6.0 ± 0.03 ^{c}	7.8 ± 0.05 ^{b}
	6.0	6.0 ± 0.07 ^{d}	6.0 ± 0.08 ^{b}	6.0 ± 0.09 ^{c}	7.6 ± 0.07 ^{c}
	6.0	6.2 ± 0.05 ^{a}	6.0 ± 0.07 ^{a}	6.1 ± 0.07 ^{a}	6.2 ± 0.07 ^{a}
S. aureus	6.0	6.0 ± 0.08 ^{b}	6.0 ± 0.01^a	6.0 ± 0.05 ^{b}	6.1 ± 0.06 ^{b}
	6.0	6.0 ± 0.03 ^{b}	6.0 ± 0.04 ^{a}	6.0 ± 0.03 ^{b}	6.0 ± 0.02 ^{c}
